# Amplifier or substitute? A systematic review of generative AI’s impact on higher-order cognitive skills among university students

**DOI:** 10.3389/fpsyg.2026.1863931

**Published:** 2026-06-22

**Authors:** Fawzia Omer Alubthane

**Affiliations:** Department of Curriculum and Instruction, College of Education, Imam Mohammad Ibn Saud Islamic University (IMSIU), Riyadh, Saudi Arabia

**Keywords:** cognitive over-reliance, creative thinking, critical thinking, dual-mechanism model, generative artificial intelligence, higher education, problem-solving, systematic review

## Abstract

**Background:**

As generative artificial intelligence (GenAI) becomes embedded in university learning environments, understanding its effects on students’ higher-order cognitive development has become one of the most pressing questions in educational research.

**Methods:**

This systematic review was conducted in accordance with PRISMA 2020 guidelines, synthesizing 89 peer-reviewed journal articles published between 2024 and 2026 from Web of Science and Scopus. Narrative synthesis was employed to integrate findings across studies.

**Results:**

GenAI’s cognitive effects were neither uniform nor unconditional: positive outcomes were documented in 40.4% of studies and mixed or conditional effects in 23.6%. ChatGPT was the predominant tool examined (*n* = 61, 68.5%), and mixed-methods designs were most prevalent (42.7%). Critically, 55.1% of studies employed no specified pedagogical strategy, and explicit theoretical frameworks were identified in only 25.8% of the corpus. Over-reliance emerged as the leading cognitive risk (33.7%), followed by reduced analytical autonomy (20.2%) and cognitive offloading (18.0%).

**Discussion:**

These findings support a Dual-Mechanism Model: GenAI functions as a cognitive amplifier under structured pedagogical conditions and as a cognitive substitute under unguided use. The evidence calls on universities to anchor GenAI integration within deliberate instructional frameworks that preserve students’ cognitive agency and foreground autonomous higher-order reasoning.

## 1 Introduction

The rapid rise of generative artificial intelligence (GenAI) has reshaped higher education in ways that few could have anticipated. Tools such as ChatGPT, Google Gemini, and other large language model-based platforms have moved from novelty to normalized academic utility within a remarkably short timeframe, prompting urgent questions about what this means for how students learn and develop cognitively (Lo, 2023; Abdallah et al., 2025).

At the heart of these questions lies a deceptively simple concern: does GenAI help or hinder the development of the higher-order cognitive skills that universities exist to cultivate—critical thinking, creative thinking, and complex problem-solving? These are not peripheral competencies. They are the foundational capacities that 21st-century labor markets demand and that democratic civic life depends upon (Rudolph et al., 2023; Sardi et al., 2025).

The stakes of this issue can be illustrated with specific examples. In critical thinking, a student evaluating the credibility of contradictory sources may use GenAI to produce a comparative summary—either as a scaffold that supports analytical judgment, if the student interrogates the output, or as a substitute for it, if the output is accepted uncritically. In creative thinking, a student composing an original argument may use GenAI as a brainstorming aid—stimulating divergent thinking if used as a source of inspiration, yet constraining originality if the ideas generated are adopted wholesale. In problem-solving, an engineering student working through a design challenge may use GenAI to model possible solutions—deepening reasoning when the student must evaluate and refine those models yet undermining it when a ready-made solution is simply extracted and applied without reflection. These three examples illustrate that the manner in which GenAI is used is inseparable from the type of cognitive outcome it produces.

The early evidence is genuinely mixed. Some studies report that GenAI enhances critical thinking by supporting knowledge exploration, metacognitive reflection, and structured inquiry (Premkumar et al., 2024; Ruiz-Rojas et al., 2024), though its effects on higher-order reasoning—evaluation, synthesis, judgment—remain contested (Essien et al., 2024). In the domain of creative thinking, the picture is similarly dual-edged: GenAI can stimulate divergent thinking and expand ideational fluency, yet may simultaneously constrain originality when students engage with it passively (Habib et al., 2024; Olaya and Bajaña, 2025).

In problem-solving, self-regulation emerges as a key mediating variable—students who use GenAI with metacognitive intentionality appear to benefit, while those who rely on it habitually may see their autonomous reasoning capacity diminish (Zhou et al., 2024; Sardi et al., 2025). These patterns are further shaped by digital literacy, academic support structures, and the mode of human-AI interaction (Kasneci et al., 2023; Agaoglu et al., 2025).

Despite the growing volume of research, the evidence base remains fragmented. Studies are frequently limited by small samples, short observation windows, and narrow disciplinary scope, and comprehensive syntheses examining all three cognitive domains jointly are notably absent (Chan and Hu, 2023; Lim et al., 2023; Ruiz-Rojas et al., 2024). This gap is consequential: without a coherent, evidence-based account of what GenAI does to higher-order cognition—and under what conditions—educators and institutions are navigating one of the most significant pedagogical transitions of the century largely without a map.

The present systematic review seeks to provide that map. By synthesizing empirical and theoretical evidence across diverse disciplinary and geographical contexts, it aims to clarify what is currently known, surface where genuine uncertainty persists, and offer a research agenda that can guide both future inquiry and the responsible integration of GenAI into university curricula.

### 1.1 Statement of the problem

The rapid integration of generative artificial intelligence (GenAI) into university learning environments has created an urgent need for a rigorous, evidence-based understanding of its effects on students’ higher-order cognitive competencies. As tools such as ChatGPT, Google Gemini, and Microsoft Copilot become embedded in academic life, a fundamental tension has emerged: while GenAI is widely promoted as a catalyst for enhanced learning and intellectual engagement, a growing body of evidence simultaneously points to its potential to erode the very competencies—critical thinking, creative thinking, and problem-solving—that lie at the heart of higher education’s developmental mission (Kasneci et al., 2023; Perkins et al., 2024; Zhai et al., 2024).

The empirical picture is genuinely contradictory. Some studies report meaningful gains in critical thinking and creative ideation when GenAI is used as a cognitive scaffold (Habib et al., 2024; Zhou et al., 2024). Others document measurable reductions in analytical independence and original thought, particularly under unstructured conditions (Essien et al., 2024; Zhai et al., 2024). In the domain of problem-solving, the relationship appears neither linear nor unconditional, but mediated by self-regulation, digital literacy, and the quality of pedagogical scaffolding (Hou et al., 2025). These contradictions reflect a deeper theoretical uncertainty: Does GenAI function as a *cognitive amplifier* or a *cognitive substitute* in university contexts?

The selection of critical thinking, creative thinking, and problem-solving as the focal cognitive domains is theoretically grounded and practically motivated. These three constructs represent the highest tiers of Bloom’s Revised Taxonomy (Anderson and Krathwohl, 2001)—the cognitive capacities most associated with graduate-level intellectual development and least amenable to automation. They are also the domains most frequently identified by employers and policy frameworks as essential for 21st-century professional competence (World Economic Forum, 2023), making their potential displacement by AI a matter of significant educational consequence.

Five interrelated research gaps compound this uncertainty. First, existing studies have examined critical thinking, creativity, and problem-solving in isolation rather than as interdependent dimensions of a unified higher-order thinking framework (Premkumar et al., 2024; Sardi et al., 2025). Second, the methodological quality of the literature remains uneven—marked by small samples, cross-sectional designs, and heavy reliance on self-reported data, with longitudinal evidence almost entirely absent (Ruiz-Rojas et al., 2024; Yusuf et al., 2024).

Third, the cognitive risk dimension—over-reliance, cognitive offloading, and the outsourcing of analytical reasoning—has received disproportionately little systematic attention despite compelling evidence of its prevalence (Perkins et al., 2024; Zhai et al., 2024). Fourth, the evidence base remains geographically skewed toward Western and East Asian contexts, with limited representation from the Global South and the Arab world (Zawacki-Richter et al., 2019; Yusuf et al., 2024). Fifth, most existing reviews lack theoretically grounded synthesis frameworks, limiting their capacity to inform pedagogical practice and institutional policy (Tlili et al., 2023).

Together, these gaps leave educational stakeholders—faculty, curriculum designers, and institutional leaders—without a coherent, evidence-based understanding of what GenAI actually does to students’ cognitive development, and under what conditions its effects are constructive rather than detrimental. The present systematic review is designed to address this problem by treating critical thinking, creative thinking, and problem-solving not as isolated variables, but as constitutive elements of the higher-order cognitive architecture that higher education is obligated to cultivate.

### 1.2 Research questions

Main Research Question: What is the state of empirical evidence on the effects of generative artificial intelligence on the development of critical thinking, creative thinking, and problem-solving skills among university students, and what conditions moderate or mediate these effects?

RQ1 (Effect & Pedagogy): What is the nature and pattern of GenAI’s impact on university students’ critical thinking, creative thinking, and problem-solving skills, and through which pedagogical strategies or instructional frameworks is this impact most constructively mediated?

RQ2 (Methodologies & Risks): What theoretical and methodological trends characterize the current empirical literature on GenAI in higher education, and what patterns of cognitive risk—such as over-reliance, reduced analytical autonomy, and cognitive offloading—emerge from these studies?

RQ3 (Gaps & Future Directions): What significant research gaps exist in the current evidence base, and what evidence-based recommendations can be offered to educators, curriculum designers, and institutional policymakers to support a balanced and pedagogically responsible integration of GenAI in university settings?

### 1.3 Theoretical framework

The current review is based on three complementary theoretical perspectives, which collectively inform the Dual-Mechanism Model proposed here. The first is Self-Regulated Learning (SRL) Theory (Zimmerman, 2002) which conceptualizes learning as a cyclical process of goal-setting, self-monitoring, and reflective evaluation. SRL provides a theoretical foundation for understanding how structured GenAI use, when prompted by metacognitive questioning, functions as a cognitive amplifier, whereas unstructured use undermines the self-regulatory processes that support independent thinking.

The second is Vygotsky’s Zone of Proximal Development (ZPD) and the scaffolding concept (Vygotsky, 1978). Here, GenAI can act as a dynamic scaffold, extending students’ cognitive reach beyond what they could achieve independently—provided the scaffolding is deliberately removed as competence is gained. When scaffolding becomes fixed, however, it may replace rather than assist cognitive development.

The third theory is Cognitive Load Theory (Sweller, 1988) which distinguishes between intrinsic load (task complexity), extraneous load (ineffective instructional design), and germane load (effort aimed at schema formation). GenAI’s capacity to reduce extraneous load can, under structured conditions, free cognitive resources for deeper reasoning. Under unguided conditions, however, it may reduce germane load alongside extraneous load—displacing the effortful processing that builds durable cognitive schemas. Together, these three frameworks explain the conditional nature of GenAI’s cognitive effects and provide the theoretical scaffolding for the Dual-Mechanism Model developed in the Discussion.

## 2 Materials and methods

### 2.1 Review design

This systematic review was conducted and reported in accordance with the Preferred Reporting Items for Systematic Reviews and Meta-Analyses 2020 guidelines (Page et al., 2021), which provide the highest standard for transparent, reproducible, and comprehensive evidence synthesis. The systematic review design was selected as the most appropriate framework for synthesizing the heterogeneous and rapidly expanding body of empirical evidence on GenAI’s effects on university students’ critical thinking, creative thinking, and problem-solving skills. To promote methodological transparency, all eligibility criteria, search strategies, and synthesis procedures were specified and documented prior to data collection, minimizing the risk of post-hoc reporting bias. This review was not prospectively registered in PROSPERO or any equivalent international register prior to commencement. This is acknowledged as a methodological limitation and is addressed further in Section 5.

### 2.2 Eligibility criteria

Eligibility criteria were established prior to the search process and operationalized using the PICO framework (Population, Intervention, Comparison, Outcome), adapted to the educational context of this review as follows:

Population (P): Undergraduate and postgraduate students, or faculty and educators, enrolled in or affiliated with accredited higher education institutions worldwide. Studies involving faculty were included where their instructional practices directly informed the pedagogical conditions mediating GenAI’s cognitive effects on students.

Intervention (I): Any generative artificial intelligence tool or platform used in academic or instructional contexts, including large language models (e.g., ChatGPT, Google Gemini, Microsoft Copilot) and AI-assisted writing or content generation tools.

Comparison (C): Studies with or without a control or comparison group were eligible, encompassing experimental, quasi-experimental, pre-post, descriptive, and correlational designs—reflecting the exploratory nature of much of the current literature in this domain.

Outcomes (O): The primary outcomes of interest were: (1) critical thinking, encompassing analysis, evaluation, logical reasoning, and reflective judgment; (2) creative thinking, including divergent thinking, ideation, originality, and creative confidence; and (3) problem-solving, comprising complex problem identification, solution generation, and decision-making. Studies addressing any one or more of these outcomes were considered eligible.

### 2.3 Inclusion criteria

Studies were included if they met all the following criteria:

- Published in a peer-reviewed journal indexed in Web of Science (Clarivate) or Scopus between January 2024 and March 2026—a period chosen to capture empirical evaluations of advanced GenAI tools rather than early exploratory perceptions.
- Focused specifically on generative AI tools, including large language models and AI-powered platforms (e.g., ChatGPT, Google Gemini, Microsoft Copilot), rather than traditional AI, adaptive learning systems, or rule-based chatbots without generative capabilities.
- Included university-level students, faculty, and educators as the primary study population, with no restrictions on discipline, year of study, or geographic location. Studies involving faculty and educators were retained where their instructional practices and perceptions directly informed the pedagogical conditions mediating GenAI’s cognitive effects on students.
- Reported empirical data or evidence-based analysis on at least one of the three target cognitive outcomes: critical thinking, creative thinking, or problem-solving.
- Published in the English language.
- Employed quantitative, qualitative, mixed-methods, or evidence-synthesis research designs, including experimental, quasi-experimental, survey-based, case study, correlational, and literature or scoping review approaches.

While systematic reviews and meta-analyses were excluded as secondary syntheses, literature reviews and scoping reviews were retained where they provided original analytical frameworks, identified research gaps, or offered evidence-based discussion directly relevant to the review’s three target cognitive domains—contributions that primary empirical studies alone cannot provide.

### 2.4 Exclusion criteria

Studies were excluded if they met any of the following criteria:

- Published before January 2024, with the exception of one 2023 study retained on the basis of its direct relevance to the review’s core outcomes.
- Focused on non-generative AI tools—such as intelligent tutoring systems, rule-based chatbots, predictive modeling, or recommendation engines—that do not possess the generative and reasoning capabilities central to this review’s focus.
- Conducted with participants below university level (primary or secondary school students).
- Consisted solely of theoretical, conceptual, opinion, or editorial content without empirical data.
- Reported outcomes unrelated to the three target cognitive domains, such as studies measuring language proficiency gains, academic performance metrics, or user satisfaction, without any reference to critical thinking, creative thinking, or problem-solving.
- Constituted duplicate publications, conference proceedings, theses, dissertations, or gray literature not subject to peer review.
- Constituted systematic reviews or meta-analyses, as these represent secondary syntheses rather than primary investigations of GenAI’s cognitive effects.
- Published in languages other than English.

Studies reporting exclusively on general user attitudes toward GenAI—without addressing cognitive outcomes, pedagogical challenges, or ethical concerns—were excluded. However, research exploring qualitative experiences, perceived barriers, and ethical dimensions of GenAI integration was retained, given that such evidence captures critical pedagogical considerations not reducible to performance metrics alone.

### 2.5 Information sources and search strategy

A systematic search was conducted across two major databases—Web of Science (Clarivate) and Scopus—selected for their comprehensive coverage of educational technology, cognitive science, and higher education research. The search was conducted in March 2026 and covered publications from January 2024 onward, with one 2023 study retained based on its direct relevance to the review’s core outcomes.

The search strategy was developed in accordance with the PRISMA-S extension for systematic search reporting (Rethlefsen et al., 2021), combining controlled vocabulary and free-text keywords linked by Boolean operators. The full search string applied across both databases was as follows:

*(“generative artificial intelligence” OR “generative AI” OR “ChatGPT” OR “large language model” OR “LLM” OR “GPT-4” OR “Gemini” OR “Copilot” OR “AI chatbot”) AND (“critical thinking” OR “higher-order thinking” OR “analytical thinking” OR “reflective thinking”) AND (“creative thinking” OR “creativity” OR “divergent thinking” OR “creative problem-solving” OR “originality”) AND (“problem-solving” OR “complex reasoning” OR “cognitive skills”) AND (“higher education” OR “university students” OR “undergraduate” OR “postgraduate” OR “tertiary education”)*

The search string was adapted to each database’s specific syntax to maximize retrieval sensitivity.

### 2.6 Study selection process

**Figure 1:**
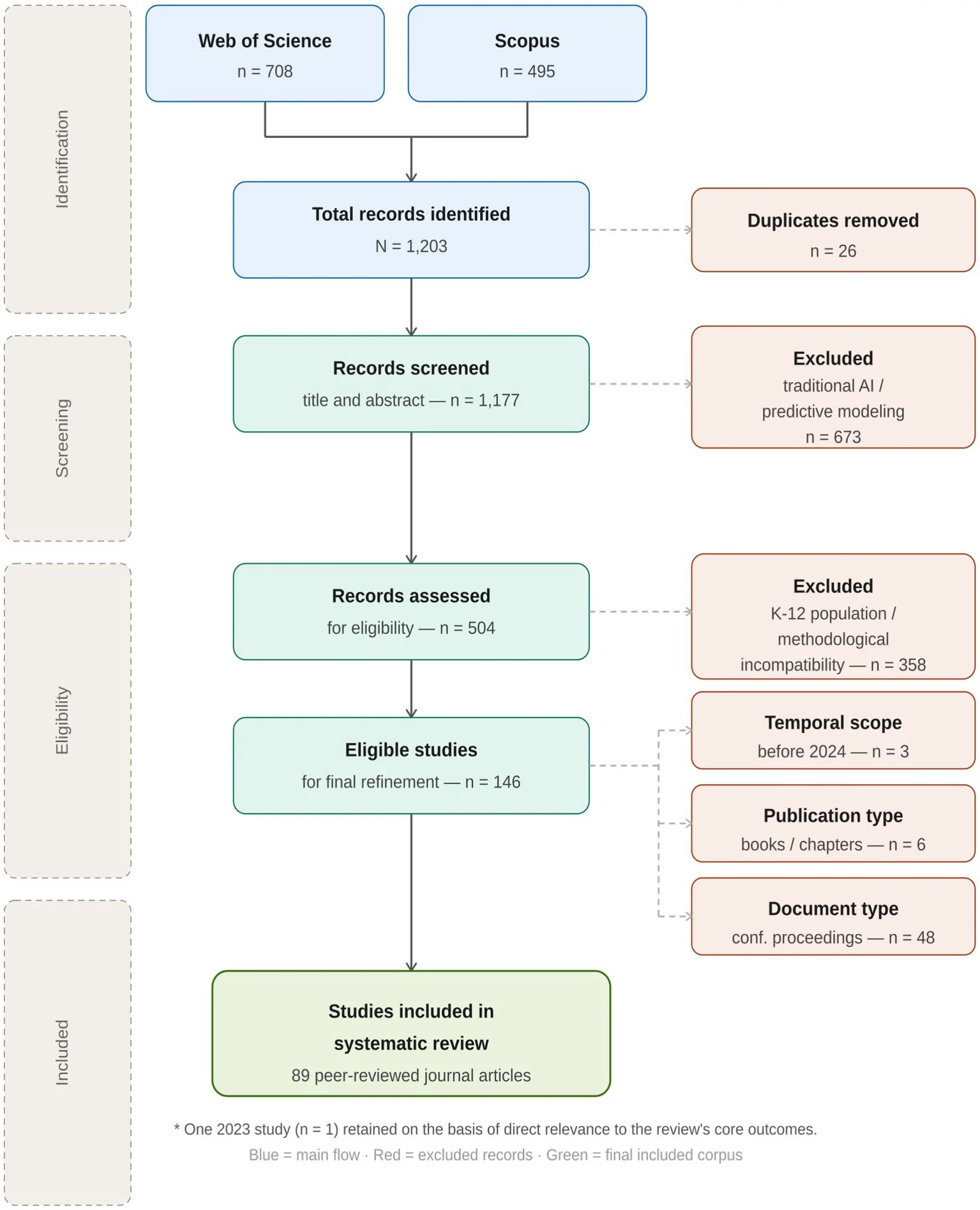
PRISMA 2020 flow diagram—study selection process (Page et al., 2021). *One 2023 study (*n* = 1) was retained based on its direct relevance to the review’s core outcomes.

The selection process followed a two-stage screening procedure conducted by the sole researcher in alignment with Page et al. (2021) recommendations. To mitigate bias inherent in single-reviewer designs, all retrieved records were first imported into Elicit for automated deduplication, then screened against the pre-established eligibility criteria in two independent passes separated by a minimum two-week interval—allowing each record to be assessed without direct recall of prior decisions (Siddaway et al., 2019). In Stage 2, records deemed potentially eligible were assessed against the complete inclusion and exclusion criteria, with reasons for exclusion systematically recorded and reported in Figure 1. To verify screening consistency, a 10% random sample of excluded records at each stage was re-screened after a two-week interval. The resulting Cohen’s Kappa coefficient of *κ* = 0.87 indicates near-perfect intra-rater agreement, consistent with recognized mitigation standards for single-author systematic reviews (Gough et al., 2017).

### 2.7 Data extraction

A standardized extraction form was developed and piloted on five studies before full implementation. To minimize error, each study was extracted twice by the same researcher with a minimum one-week interval between sessions, and any discrepancies were resolved before synthesis.

The form captured the following for each study: bibliographic details (authors, year, journal, and DOI); study design and methodology; GenAI tool examined and platform; cognitive outcomes measured across the three target domains—critical thinking, creative thinking, and problem-solving—along with key findings, effect sizes, and statistical indicators where reported; pedagogical strategies and cognitive risk patterns; theoretical frameworks underpinning each study; and research gaps and recommendations identified by the original authors. Where available in the abstract-level data, participant characteristics—including sample size, academic level, discipline, and geographic context—were additionally recorded. Given that full-text access was restricted for a subset of studies, certain characteristics could not be verified independently and are reported as “not reported in abstract.

### 2.8 Quality assessment

Each included study was appraised using a tool matched to its methodological design: RoB 2 (Sterne et al., 2019) for randomized controlled trials, ROBINS-I (Sterne et al., 2019) for quasi-experimental designs, MMAT version 2018 (Hong et al., 2018) for survey-based and mixed-methods studies, and the CASP Qualitative Checklist (Critical Appraisal Skills Programme (CASP), n.d.) for qualitative research. To strengthen consistency within a single-reviewer design, 15% of included studies were re-appraised after a minimum two-week interval. Quality scores were not used as grounds for exclusion; rather, they informed how confidently findings were interpreted and helped identify methodological limitations that may explain variability across studies—an approach consistent with best practice in educational systematic reviews (Gough et al., 2017). Overall, the majority of included studies were assessed as meeting acceptable quality thresholds for their respective designs—a finding consistent with the corpus’s restriction to peer-reviewed journals indexed in Web of Science (Clarivate) and Scopus, both of which apply rigorous indexing standards that serve as a baseline quality filter. Methodological limitations—principally cross-sectional designs, small samples, and self-reported measures—were nonetheless noted across a substantial proportion of the corpus.

### 2.9 Synthesis approach

Given the diversity of study designs, outcome measures, and GenAI tools across the corpus, a narrative synthesis was adopted following Popay et al., (2006) and the PRISMA 2020 framework (Page et al., 2021). A statistical meta-analysis was not appropriate, as outcome instruments varied considerably, and effect sizes were rarely reported in a standardized form. The synthesis was organized around the three target cognitive domains—critical thinking, creative thinking, and problem-solving—with cross-cutting attention to theoretical frameworks, methodological trends, pedagogical strategies, and geographic patterns. Where studies combined quantitative and qualitative data, findings were integrated to build a fuller picture of each outcome. Thematic tabulation and textual description were used throughout to surface patterns and account for heterogeneity across studies.

## 3 Results

### 3.1 Study selection process

The selection process followed the PRISMA 2020 guidelines to ensure transparency and rigor. Initially, a total of 1,203 records were identified from Web of Science (*n* = 708) and Scopus (*n* = 495). After removing 26 duplicate records, 1,177 unique studies remained for title and abstract screening. In the initial screening phase, 673 records were excluded as they did not focus on Generative AI (GenAI), instead covering traditional AI applications or predictive modeling. The remaining 504 studies underwent eligibility assessment, during which 358 studies were excluded on the basis of inappropriate study population (K-12 or pre-university settings) or methodological incompatibility with the inclusion criteria. For the final refinement of the 146 eligible studies, specific quality and temporal criteria were applied. A total of 57 studies were excluded, comprising: (1) Temporal scope (*n* = 3): studies published before 2024 were excluded to focus on the most recent evidence base; one 2023 study was retained on the basis of its direct relevance to the review’s core outcomes; (2) Publication type (*n* = 6): books and book chapters were excluded to maintain focus on peer-reviewed research; and (3) Document type (*n* = 48): conference proceedings were excluded to prioritize high-impact, full-length journal articles. Ultimately, 89 peer-reviewed journal articles were included in the final systematic review for data extraction and qualitative synthesis. A complete list of the 89 peer-reviewed studies included in this systematic review are provided in Supplementary material. Table 1 and Figure 1 illustrate the study selection process.

**Table 1:** Summary of the systematic study selection process (PRISMA 2020).

PRISMA stage	Description and criteria	*n*
1. Identification	Records identified from Web of Science	708
	Records identified from Scopus	495
	Total initial records identified	1,203
	Duplicates removed	(26)
2. Screening	Records screened (title and abstract)	1,177
	Records excluded (traditional AI/predictive modeling)	(673)
3. Eligibility	Records assessed for eligibility	504
	Records excluded (inappropriate population or methodological incompatibility)	(358)
	Eligible studies for final refinement	146
4. Final Refinement	Total records excluded (quality/temporal criteria)	(57)
	— Temporal scope (studies published before 2024, excl. One retained)	(3)
	— Publication type (books and book chapters)	(6)
	— Document type (conference proceedings)	(48)
5. Included	Final studies included in systematic review	89

One study published in 2023 (*n* = 1) was retained based on its direct relevance to the review’s core outcomes. Figures in parentheses indicate excluded records. PRISMA = Preferred Reporting Items for Systematic Reviews and Meta-Analyses (Page et al., 2021).

### 3.2 Description of included studies

Table 2 presents the characteristics of the 89 included studies. The corpus spans 2024–2026, with the majority published in 2025 (*n* = 49, 55.1%), followed by 2026 (*n* = 22, 24.7%) and 2024 (*n* = 17, 19.1%), confirming the recency of this research domain. Geographically, most studies were conducted in multi-national or unspecified contexts (*n* = 60, 67.4%), with the Global South representing the largest identifiable regional cluster (*n* = 17, 19.1%). Mixed-methods designs were the most prevalent approach (*n* = 38, 42.7%), followed by quantitative designs (*n* = 33, 37.1%) and qualitative designs (*n* = 10, 11.2%). ChatGPT was the predominant tool examined (*n* = 61, 68.5%), reflecting its dominant position in academic adoption during the review period.

**Table 2:** Description of included studies (*N* = 89).

Category	Classification	*n*	%
Publication year	2025	49	55.1%
	2026	22	24.7%
	2024	17	19.1%
	2023 (retained)	1	1.1%
Geographic region	Multi-national/Unspecified	60	67.4%
	Global South	17	19.1%
	East Asia	5	5.6%
	North America	3	3.4%
	Europe/Turkey	3	3.4%
	Latin America	2	2.2%
Methodological design	Mixed-Methods	38	42.7%
	Quantitative	33	37.1%
	Qualitative	10	11.2%
	Not reported in abstract	6	6.7%
	Review/Bibliometric	2	2.2%
GenAI tool examined	ChatGPT (various versions)	61	68.5%
	General GenAI (unspecified platform)	28	31.5%
	Microsoft Copilot	2	2.2%
	Google Gemini	1	1.1%
Academic level	Undergraduate	25	28.1%
	University students (unspecified level)	18	20.2%
	Not reported in abstract	17	19.1%
	Faculty/Educators	13	14.6%
	Both UG & PG	11	12.4%
	Postgraduate	5	5.6%
Academic discipline	Language/EFL/ESL	45	50.6%
	STEM	16	18.0%
	General/Multi-disciplinary	13	14.6%
	Business/Management	6	6.7%
	Social Sciences/Humanities	5	5.6%
	Health Sciences	2	2.2%
	Teacher Education	1	1.1%
	Law	1	1.1%
Theoretical framework	Without framework/not reported	66	74.2%
	With identifiable framework	23	25.8%
	— Established theories (TAM, UTAUT2, SCT.)	14	15.7%
	— Design/methodological frameworks	9	10.1%

Studies may appear in more than one GenAI tool sub-category; consequently, tool frequencies sum to more than *N* = 89. “Not reported in abstract” indicates that the characteristic was not determinable from abstract-level data. Theoretical framework sub-categories are not mutually exclusive.

Disciplinarily, Language and EFL/ESL contexts dominated the corpus (*n* = 45, 50.6%), followed by STEM (*n* = 16, 18.0%) and General or Multi-disciplinary settings (*n* = 13, 14.6%). Undergraduate students constituted the most frequently studied population (*n* = 25, 28.1%), while Faculty and Educators were the focus of 13 studies (14.6%). Regarding theoretical grounding, explicit frameworks were identified in only 23 studies (25.8%)—most notably TAM (*n* = 4, 4.5%) and UTAUT/UTAUT2 (*n* = 3, 3.4%)—while 74.2% of the corpus either stated the absence of a theoretical framework or did not report one.

### 3.3 Results by research question

#### 3.3.1 Direction and pattern of impact (RQ1)

Of the 89 included studies, 72 (80.9%) reported a discernible direction of impact on at least one target cognitive domain. Positive effects were documented in 36 studies (40.4%), mixed or conditional effects in 21 (23.6%), and negative effects in 15 (16.9%). The remaining 17 studies (19.1%) did not directly measure cognitive impact—focusing instead on perceptions, scale development, tool performance evaluation, or pedagogical framework design. Critical thinking was the most frequently targeted domain (*n* = 68, 76.4%), followed by problem-solving (*n* = 56, 62.9%) and creative thinking (*n* = 32, 36.0%).

##### 3.3.1.1 Impact on critical thinking

Evidence on critical thinking was conditional and context-dependent. Oliva-Córdova et al. (2025) reported significant improvements across Bloom’s Revised Taxonomy levels following structured GenAI integration, and Chen et al. (2025) demonstrated that ChatGPT-supported argumentation tasks enhanced critical reasoning. Conversely, Gammoh (2024) identified diminished critical thinking as a primary risk of unguided ChatGPT use, while Chea and Deng (2026) documented a paradox whereby GenAI simultaneously enhanced creativity yet eroded critical thinking through passive consumption of AI-generated content.

##### 3.3.1.2 Impact on creative thinking

Creative thinking yielded a moderately positive signal, tempered by risks of idea homogenization. Urban et al. (2024) reported significant gains in solution quality (*d* = 0.69), elaboration (*d* = 0.61), and originality (*d* = 0.55) in a randomized design, and Meng et al. (2025) demonstrated that a GenAI literacy-integrated instructional approach improved creative and critical thinking among pre-service teachers. However, Wang et al. (2026) cautioned that while aggregate creative performance improved, convergence in solution approaches risked inhibiting genuinely original thought.

##### 3.3.1.3 Impact on problem-solving

Problem-solving produced the most consistently positive empirical signal. Zhou et al. (2025) demonstrated that ChatGPT-facilitated scaffolding significantly improved mathematical problem-solving, and Tu et al. (2026) showed that AI-generated feedback integrated with knowledge graph technology enhanced problem-solving through reduced cognitive load and improved self-monitoring. Xiao and Hu (2026) identified a non-linear relationship between ChatGPT use frequency and performance, suggesting an inverted-U pattern in which moderate over-reliance temporarily depresses autonomous problem-solving capacity.

##### 3.3.1.4 Pedagogical mediation

A critical finding concerns the centrality of pedagogical scaffolding. Only 40 studies (44.9%) reported a specific instructional strategy, while 49 (55.1%) reported no specific strategy—a figure that reflects methodological heterogeneity rather than pedagogical absence alone, as review studies, qualitative explorations of naturalistic use, and studies where full-text access was unavailable collectively account for a portion of this category.

Among quantitative and mixed-methods studies specifically, 41 of 71 (57.7%) reported no structured strategy, a proportion that nonetheless confirms structured pedagogical integration as the exception rather than the norm. The evidence consistently indicates that structured integration—through scaffolding, argumentation, or reflective cycles—produces more positive and durable cognitive outcomes than unguided use.

Among identified strategies, reflective learning (*n* = 11, 12.4%) and collaborative learning (*n* = 7, 7.9%) were most prevalent. The evidence consistently indicates that structured integration—through scaffolding, argumentation, or reflective cycles—produces more positive and durable cognitive outcomes than unguided use. The results for RQ1 are presented in Table 3.

**Table 3:** RQ1—impact direction and pedagogical strategies (*N* = 89).

Category	Classification	*n*	%
Impact direction	Positive	36	40.4%
	Mixed/Conditional	21	23.6%
	Negative	15	16.9%
	Not directly measured	17	19.1%
Cognitive domain targeted	Critical Thinking	68	76.4%
	Problem-Solving	56	62.9%
	Creative Thinking	32	36.0%
Pedagogical strategy	Without specific strategy	49	55.1%
	With specific strategy	40	44.9%
	— Reflective Learning	11	12.4%
	— Collaborative Learning	7	7.9%
	— Scaffolding/PBL	8	9.0%
	— Argumentation/AI Feedback	6	6.7%
	— Other strategies	4	4.5%

Cognitive domain percentages exceed 100% as studies addressed multiple domains simultaneously. “Not directly measured” includes studies focused on perceptions, scale development, tool performance evaluation, and pedagogical framework design; seven adoption/policy studies are subsumed within this category. Pedagogical strategy sub-categories are not mutually exclusive. The 49 studies with no specific strategy include review/bibliometric (*n* = 2), qualitative (*n* = 10), and abstract-only studies (*n* = 6) for which strategy verification was limited or not applicable.

#### 3.3.2 Methodologies and risks (RQ2)

##### 3.3.2.1 Theoretical and methodological landscape

Explicit theoretical frameworks were identified in only 23 studies (25.8%), with TAM (*n* = 4, 4.5%) and UTAUT/UTAUT2 (*n* = 3, 3.4%) the most prevalent. The remaining 74.2% either stated the absence of a framework or did not report one. Methodologically, mixed-methods designs dominated (*n* = 38, 42.7%), followed by quantitative (*n* = 33, 37.1%) and qualitative (*n* = 10, 11.2%) approaches. Longitudinal designs and randomized controlled trials with large samples were notably absent, constraining causal inference across the corpus.

##### 3.3.2.2 Cognitive risk patterns

Thirty-eight studies (42.7%) documented cognitive risks. Over-reliance was the most prevalent (*n* = 30, 33.7%), followed by reduced analytical autonomy (*n* = 18, 20.2%), reduced critical thinking (*n* = 18, 20.2%), and cognitive offloading (*n* = 16, 18.0%). These risks clustered predominantly in studies where GenAI use was unstructured, providing empirical grounding for the dual-mechanism model proposed in Section 4. The theoretical, methodological, and risk findings for RQ2 are summarized in Table 4.

**Table 4:** RQ2—theoretical frameworks and cognitive risk patterns (*N* = 89).

Category	Classification	*n*	%
Theoretical Framework	Without framework/not reported	66	74.2%
	With identifiable framework	23	25.8%
	— TAM	4	4.5%
	— UTAUT/UTAUT2	3	3.4%
	— Constructivism	1	1.1%
	— Cognitive Load Theory	1	1.1%
	— Other frameworks	14	15.7%
Methodological design	Mixed-Methods	38	42.7%
	Quantitative	33	37.1%
	Qualitative	10	11.2%
	Not reported in abstract	6	6.7%
	Review/Bibliometric	2	2.2%
Cognitive risks	Studies reporting ≥1 risk	38	42.7%
	— Over-reliance	30	33.7%
	— Reduced Analytical Autonomy	18	20.2%
	— Reduced Critical Thinking	18	20.2%
	— Cognitive Offloading	16	18.0%
	— Academic Integrity concerns	3	3.4%
	— Metacognitive Laziness	2	2.2%
	— AI Inaccuracy/Hallucination	2	2.2%

“Other frameworks” includes Design-Based Research, Legitimation Code Theory, Genre-Based Pedagogy, Postdigital Theory, Ecopedagogy, Collaborative Action Research, Foresight 3.0, and seven additional frameworks each appearing in one study. Cognitive risk categories are not mutually exclusive.

#### 3.3.3 Research gaps and recommendations (RQ3)

Forty-eight studies (53.9%) explicitly identified research gaps. The most frequently articulated priorities were ethical frameworks (*n* = 28, 31.5%), strategies to preserve critical thinking (*n* = 21, 23.6%), and policy or institutional guidelines (*n* = 12, 13.5%). Teacher training (*n* = 6, 6.7%), longitudinal and larger-sample designs (*n* = 6, 6.7%), and diverse cultural contexts (*n* = 3, 3.4%) rounded out the gap landscape. These priorities collectively signal a field transitioning from descriptive documentation toward normative and prescriptive inquiry. The research gaps and recommendations identified across the corpus are presented in Table 5.

**Table 5:** RQ3—research gaps and evidence-based recommendations (*N* = 89).

Category	Classification	*n*	%
Studies with gaps/recs	Yes	48	53.9%
	No/Not specified	41	46.1%
Research gap identified	Ethical frameworks needed	28	31.5%
	Strategies to preserve critical thinking	21	23.6%
	Policy/institutional guidelines	12	13.5%
	Teacher training/faculty development	6	6.7%
	Longitudinal studies/larger samples	6	6.7%
	Diverse cultural/geographic contexts	3	3.4%

Gap categories are not mutually exclusive; studies may identify more than one gap. “Longitudinal studies/larger samples” combines two related methodological priorities each identified in three studies.

## 4 Discussion

The present review extends the work of Helal et al. (2025), whose DI-GAI-CT framework advances understanding of GenAI’s effects on critical thinking, by synthesizing all three higher-order cognitive domains jointly, grounding the dual-mechanism model in empirical patterns drawn from 89 primary studies, and documenting the specific pedagogical conditions under which each mechanism operates.

The central finding—that GenAI functions as a cognitive amplifier under structured conditions and as a cognitive substitute under unguided use—is theoretically anchored in two complementary frameworks. From a Vygotskian perspective, scaffolding produces genuine developmental gains only when calibrated to the learner’s capacity and embedded within deliberate instructional design (McPhee and Jerowsky, 2025; Rana et al., 2025). When this calibration is absent, the tool risks fostering what Fan et al. (2025) termed metacognitive laziness—the progressive outsourcing of analytical effort that yields short-term gains at the cost of durable cognitive formation. Self-regulated learning theory (Zimmerman, 2002) frames this dynamic precisely: metacognitive monitoring transforms any learning tool into a developmental resource rather than a cognitive crutch—a pathway (Zhou et al., 2024) confirmed empirically by demonstrating that self-regulation significantly mediates the relationship between GenAI use and critical thinking outcomes.

**Figure 2:**
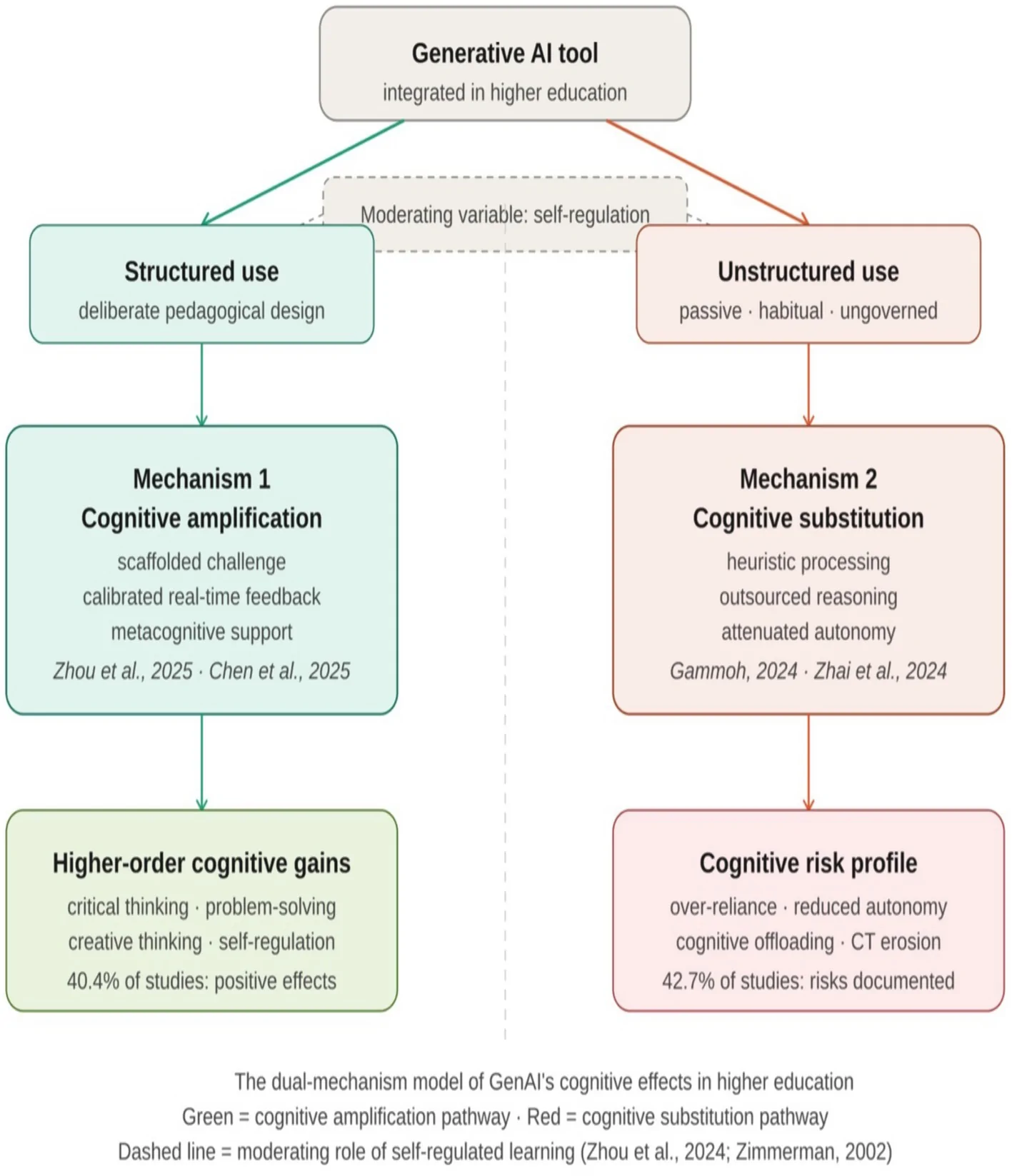
The dual-mechanism model of GenAI’s cognitive effects in higher education.

These two mechanisms operate through distinct pathways. Under Cognitive Amplification, deliberate instructional frameworks that promote active reasoning and collaborative knowledge construction extend students’ cognitive reach—as evidenced in scaffolded problem-solving (Zhou et al., 2025), argumentation-based writing (Chen et al., 2025), and reflective learning cycles (Meng et al., 2025). Under Cognitive Substitution, passive use without metacognitive guidance displaces the mental effort required for deep processing, progressively attenuating analytical autonomy and creative independence—as documented in studies of over-reliance (Gammoh, 2024; Chea and Deng, 2026) and cognitive offloading (Zhai et al., 2024; Rana et al., 2025). The cognitive risks documented here are not inherent to the technology but to the conditions of its use—a distinction the dual-mechanism model captures in its two constituent pathways (see Figure 2).

What makes this finding practically consequential is the evidence that the substitution mechanism is not inevitable. Hong et al. (2025) demonstrated that deliberately structured cognitive offload instruction—assigning lower-order tasks to GenAI while demanding evaluative engagement from students—produced greater critical thinking gains than conventional instruction. The same offloading mechanism that constitutes a risk under unguided conditions can be strategically redesigned as a pedagogical asset under deliberate instruction.

The field’s theoretical underdevelopment remains its most consequential structural limitation. Explicit frameworks were identifiable in only 25.8% of the corpus—an epistemological gap that constrains the cumulative generativity of the literature. It should be noted, however, that for a subset of studies where only abstract-level data were accessible, the absence of a reported framework may reflect retrieval constraints rather than genuine theoretical absence. As Yan et al. (2024) argued that translating GenAI’s promise into durable cognitive outcomes demands theoretical rigor alongside empirical breadth. The dominance of technology acceptance models among theoretically grounded studies signals a field still asking whether students *adopt* GenAI, rather than what that adoption does to their cognitive architecture over time—and closing this gap through longitudinal, theoretically grounded designs is the defining methodological challenge facing the field.

## 5 Limitations

This review is subject to several limitations that should be considered when interpreting its findings. As a single-researcher design, structured mitigation protocols were implemented—including dual-pass screening, time-delayed re-appraisal of a 15% random subsample, and two-occasion data extraction—yet these cannot fully replicate the inter-rater reliability achievable in multi-reviewer designs. The review was not registered in PROSPERO prior to data collection; given the rapidly evolving nature of the GenAI education landscape and the intentionally narrow temporal scope of 2024–2026, prospective registration was not pursued, as the protocol was fully documented, and the review timeline did not permit pre-registration without compromising the currency of the evidence base.

Restriction to English-language publications indexed in Web of Science and Scopus may have introduced publication bias, excluding relevant evidence from non-English scholarly traditions. The narrow temporal window, while intentional, limits longitudinal generalizability and precludes conclusions about longer-term cognitive trajectories. Finally, eligibility decisions for a subset of studies were based on abstract-level data where full-text access was restricted; while this did not affect any inclusion decision—all retained studies met eligibility criteria as confirmed from their abstracts—it limited the completeness of certain extracted characteristics, reported accordingly as “not reported in abstract” in Table 2. These limitations notwithstanding, the review’s systematic protocol, breadth of corpus, and theoretically grounded synthesis framework provide a foundation for the conclusions drawn.

Finally, the review protocol was not prospectively registered in PROSPERO prior to commencement, which may introduce the possibility of selective reporting. This limitation is common in rapid systematic reviews conducted within constrained timeframes and does not affect the transparency of the search strategy or selection process, both of which are fully reported herein.

## 6 Conclusion

The central question this review pursued—what does GenAI do to the cognitive architecture of university students?—cannot be answered by pointing to a tool. It can only be answered by examining what surrounds the tool: the instructional intentions embedded in its use, the metacognitive demands placed on the learner, and the assessment cultures that signal what kind of thinking the institution actually values. The evidence from 89 studies converges on a finding that is at once empirically grounded and theoretically demanding: GenAI is cognitively neutral in the abstract and cognitively consequential in practice. Its effects are not properties of the technology; they are properties of the pedagogical conditions under which it is encountered.

This conclusion carries a deeper implication that the field has not yet fully reckoned with. The cognitive risks documented in this review—over-reliance, attenuated autonomy, the progressive outsourcing of analytical effort—are not aberrations produced by careless students or negligent institutions. They are rational adaptations to environments that reward outputs over processes, efficiency over struggle, and fluent answers over uncertain inquiry. If universities continue to assess AI-augmented products while claiming to measure human thinking, they will produce graduates who have learned to simulate the appearance of higher-order cognition without necessarily developing its substance. Redesigning assessment to foreground epistemic process—to make reasoning visible, revisable, and independently defensible—is not a technical adjustment; it is the most consequential pedagogical decision available to institutions navigating this transition.

Two structural gaps in the current evidence base demand urgent attention. The near-total absence of longitudinal research means we do not yet know what sustained, structured GenAI use does to cognitive development over time—whether it builds the metacognitive scaffolding that transfers to independent thought, or quietly erodes the tolerance for intellectual difficulty that deep learning requires. And the evidence base’s geographic concentration in East Asian and multi-national contexts means that the guidance produced by this literature may be calibrated for the most resourced educational environments and applied indiscriminately across the least resourced ones—a mismatch with profound equity implications.

These deficiencies gain heightened significance when considered within the framework of sustainable learning. This concept transcends immediate academic achievements, focusing instead on the enduring maintenance of cognitive independence, the adaptability of intellectual proficiencies across diverse situations, and the equitable cultivation of individuals capable of autonomous thought in an environment increasingly influenced by artificial intelligence. Consequently, sustainable higher education cannot be solely evaluated based on students’ performance with AI support; rather, it must assess whether such assistance enhances their capabilities, critical awareness, and intellectual fortitude compared to a scenario devoid of AI intervention.

What this review ultimately affirms is that higher education has never faced a pedagogical challenge quite like this one: a tool capable of performing many of the cognitive operations that universities exist to develop, available to every student, impossible to uninvent. The question is not whether to integrate GenAI—that decision has already been made, institution by institution, student by student. The question is whether universities will design that integration with the deliberateness it demands. Those that do will find GenAI to be what the evidence shows it can be: a genuine amplifier of human cognitive capacity. Those that do not will find it to be something more troubling—a highly capable substitute for the very thinking they were built to cultivate.

## Data Availability

The original contributions presented in the study are included in the article/Supplementary material, further inquiries can be directed to the corresponding author.
