# Supplementary material for: Amplifier or substitute? A systematic review of generative AI’s impact on higher-order cognitive skills among university students
Source: Front Psychol. 2026 Jun 22;17:1863931. doi: 10.3389/fpsyg.2026.1863931 (PMC13334428; doi:10.3389/fpsyg.2026.1863931)
Supplement: Supplementary file 1 [file Table_1.DOCX]

Supplementary Material

# Supplementary Table 1: List of Included Studies (n=89)

The following 89 peer-reviewed studies constitute the complete corpus of studies included in the systematic review.

Alba, C., Mcilwain, C., & An, R. (2026). ChatGPT on campus: how top US universities govern generative AI across higher education. Policy Reviews in Higher Education, 1-30.

Alexander, K., & Savvidou, C. (2025). Reflecting on Chapelle's view of open GenAI's role in language education: Using ChatGPT for academic writing courses in higher education. Language Teaching Research Quarterly, 51(Special Issue), 203-225. <https://doi.org/10.32038/ltrq.2025.51.02>

Alkamel, M. A. A., & Alwagieh, N. A. S. (2024). Utilizing an adaptable artificial intelligence writing tool (ChatGPT) to enhance academic writing skills among Yemeni university EFL students. Social Sciences and Humanities Open, Article 101095. <https://doi.org/10.1016/j.ssaho.2024.101095>

Almahasees, Z., Al-Natour, M., Bataineh, K. A., & Aminzadeh, S. (2024). Students' perceptions of the benefits and challenges of integrating ChatGPT in higher education. Pakistan Journal of Life & Social Sciences, 22(2). <https://doi.org/10.57239/PJLSS-2024-22.2.00256>

Alzubaidi, K. (2025). The role of generative AI in higher education: Institutional guidelines, generational gaps, and the Grok 4 challenge. Arab World English Journal (AWEJ) Special Issue on CALL, 11, 1–4. <https://doi.org/10.24093/awej/call11.1A>

Apridayani, A., Wichaidith, A., & Doromae, R. (2025). ChatGPT for English writing: A qualitative inquiry among English major students at Thai higher education. The New English Teacher, 19(2), 72. <https://search.proquest.com/openview/5c64a42ff4efd0b8e276f89fcaf77d26/1?pq-origsite=gscholar&cbl=4531125>

Argüelles-Álvarez, I. (2025). Exploring peer assessment in academic writing with ChatGPT: Insights from the Legitimation Code Theory in higher education. Teaching English with Technology, 25(1), 41-59. <https://doi.org/10.56297/vaca6841/OZFH1876/PMRR6657>

Athanassopoulos, S., Tzavara, A., Aravantinos, S., Lavidas, K., Komis, V., & Papadakis, S. (2026). Teacher education students' practices, benefits, and challenges in the use of generative AI tools in higher education. Education Sciences, 16(2), Article 228. <https://doi.org/10.3390/educsci16020228>

Bai, Y. Q. (2026). An experimental study on the impact of generative AI on university students' emotions and performance in creative problem-solving tasks. Learning and Instruction, Article 102316. <https://doi.org/10.1016/j.learninstruc.2026.102316>

Bernardi, M. L., Capone, R., Faggiano, E., & Rocha, H. (2025). Generative AI in mathematics education: Pre-service teachers' knowledge and implications for their professional development. International Journal of Mathematical Education in Science and Technology, 56(8), 1513-1530. <https://doi.org/10.1080/0020739X.2025.2490104>

Buripakdi, A. (2024). “Why Can’t I Use ChatGPT for My Academic Learning?” Voices from Ph. D. Students in a Thai University. LEARN Journal: Language Education and Acquisition Research Network, 17(2), 299-319. https://doi.org/10.70730/VRVS6900

Cena, E., McParland, A., Toivo, W., Dalton, B., Mundy, M., O'Connor, P. A., Robertson, A. E., Swingler, M., Wilson, P., & Duncan, C. (2026). Studying with GenAI: Student views on the opportunities and risks of GenAI in higher education. Education and Information Technologies, 1-27. Education and Information Technologies. <https://doi.org/10.1007/s10639-026-13923-3>

Chea, P., & Deng, F. (2026). Assets and threats of generative AI in higher education: Exploring Chinese postgraduate students' perceptions. Innovative Higher Education, 1-48. <https://doi.org/10.1007/s10755-026-09886-5>

Chen, X. Y., Liu, J. G., Liu, Y., Xiang, H. B., Guo, X. D., Cai, X. B., & Wang, X. W. (2026). Can ChatGPT outperform college students in critical thinking skills during argumentation activities? A case study. Thinking Skills and Creativity, Article 102166. <https://doi.org/10.1016/j.tsc.2026.102166>

Chen, X. Y., Jia, B. Y., Peng, X. Y., Zhao, H. C., Yao, J. J., Wang, Z., & Zhu, S. H. (2025). Effects of ChatGPT and argument map (AM)-supported online argumentation on college students' critical thinking skills and perceptions. Education and Information Technologies, 30(12), 17623-17658. <https://doi.org/10.1007/s10639-025-13471-2>

Choi, W. Y. (2026). Integrating generative AI in university mathematics: Reflective and collaborative learning as pedagogical safeguards. Education and Information Technologies,1-22. <https://doi.org/10.1007/s10639-026-13945-x>

Comerio, G. (2024). Graduate attributes in the age of AI: Suggestions for purposeful uses of ChatGPT in higher education. International Journal of Information and Learning Technology , 43(2), 141-154. <https://doi.org/10.1108/IJILT-11-2024-0268>

de la Puente, M., Torres, J., Troncoso, A. L. B., Meza, Y. Y. H., & Carrascal, J. X. M. (2024). Investigating the use of ChatGPT as a tool for enhancing critical thinking and argumentation skills in international relations debates among undergraduate students , 11(1), 55. Smart Learning Environments. <https://doi.org/10.1186/s40561-024-00347-0>

de la Puente Pacheco, M. A., Torres, J., Blanco Troncoso, A. L., Guzmán Murillo, H. J., & Carrascal, J. X. M. (2025). Enhancing critical thinking and argumentation skills in Colombian undergraduate diplomacy students: ChatGPT-assisted and traditional debate methods. Journal of Political Science Education , 21(4), 728-738.. <https://doi.org/10.1080/15512169.2025.2449936>

Scientific, L. L. (2024). Comparative Study of Local Wisdom Comprehension in Short Stories Between College Students and Ai Chatbots (Chatgpt and Gemini). Journal of Theoretical and Applied Information Technology, 102(18). https://www.jatit.org/volumes/Vol102No18/7Vol102No18.pdf

Eddine, R. J., Gide, E., & Al-Sabbagh, A. (2025). Generative AI in higher education: A cross-sector analysis of ChatGPT's impact on STEM, social sciences, and healthcare. STEM Education, 5(5), 757-801. <https://doi.org/10.3934/steme.2025035>

Essel, H. B., Vlachopoulos, D., Essuman, A. B., & Amankwa, J. O. (2024). ChatGPT effects on cognitive skills of undergraduate students: Receiving instant responses from AI-based conversational large language models (LLMs). Computers and Education: Artificial Intelligence, 6, Article 100198. <https://doi.org/10.1016/j.caeai.2023.100198>

Farhat, Z. (2025). Hooked on help: Student overreliance on ChatGPT in higher education. Journal of Information Technology Teaching Cases. <https://doi.org/10.1177/20438869251397953>

Fuller, M., & Barnes, N. (2024). The impact of ChatGPT on teaching and learning in higher education: Exploring the dual perspectives of participants who were students and teachers. New Directions for Higher Education, 2024(207), 31-46. <https://doi.org/10.1002/he.20507>

Gammoh, L. A. (2024). ChatGPT in academia: Exploring university students' risks, misuses, and challenges in Jordan. Journal of Further and Higher Education, 48(6), 608–624. <https://doi.org/10.1080/0309877X.2024.2378298>

Gammoh, L. A. (2025). ChatGPT risks in academia: Examining university educators' challenges in Jordan. Education and Information Technologies, 30, 3645–3667. <https://doi.org/10.1007/s10639-024-13009-y>

George-Reyes, C. E., Avello-Martínez, R., & Buenestado-Fernández, M. (2025). Perceptions of ChatGPT and the complexity of its impact among higher education students: Evidence across ten countries of Latin America and Europe. Educational Process: International Journal, 15, e2025171. <https://doi.org/10.22521/edupij.2025.15.171>

Getenet, S. (2024). Pre-service teachers and ChatGPT in multistrategy problem-solving: Implications for mathematics teaching in primary schools. International Electronic Journal of Mathematics Education, 19(1). <https://doi.org/10.29333/iejme/14141>

Gregory, R. W., & Narang, S. (2024). AI for learning unleashed: Pioneering generative AI in education at the University of Miami. Journal of Information Technology Teaching Cases, 20438869241266258. <https://doi.org/10.1177/20438869241266258>

He, S. Y., & Shen, Y. (2025). Learn to question: A study on the pattern of student-GenAI collaborative learning in higher education. Frontiers of Education in China, 20(4), 450.. <https://doi.org/10.3868/s110-020-025-0024-1>

Holzmann, U., Anand, S., & Payumo, A. Y. (2024). The ChatGPT fact-check: Exploiting the limitations of generative AI to develop evidence-based reasoning skills in college science courses. Advances in Physiology Education, 49(1), 191-196. <https://doi.org/10.1152/advan.00142.2024>

Hönigsberg, S., Watkowski, L., & Drechsler, A. (2025). Generative artificial intelligence in higher education: Mediating learning for literacy development. Communications of the Association for Information Systems,  56(1), 35.<https://doi.org/10.17705/1CAIS.05640>

Hou, C. Y., Zhu, G. X., Sudarshan, V., Lim, F. S., & Ong, Y. S. (2025). Measuring undergraduate students' reliance on generative AI during problem-solving: Scale development and validation. Computers & Education, 234, Article 105329. <https://doi.org/10.1016/j.compedu.2025.105329>

Hsiao, C. H., & Tang, K. Y. (2025). Beyond acceptance: An empirical investigation of technological, ethical, social, and individual determinants of GenAI-supported learning in higher education. Education and Information Technologies, 30(8), 10725-10750. <https://doi.org/10.1007/s10639-024-13263-0>

Hu, Y., Chen, J., & Hwang, G. J. (2025). A ChatGPT-supported QIVE model to enhance college students' learning performance, problem-solving and self-efficacy in art appreciation. Interactive Learning Environments, 34(2), 513-526. <https://doi.org/10.1080/10494820.2025.2503231>

Hua, J., & Cunningham, J. (2025). Two years after ChatGPT: A thematic analysis of first-year students' reflections on AI tool use in higher education. Journal of Information, Communication and Ethics in Society, 1-16. <https://doi.org/10.1108/JICES-05-2025-0118>

Jogezai, N. A., Baloch, F. A., Jaffar, M., & Khilji, G. (2025). Generative AI in higher education: University faculty perspectives on opportunities and challenges. Turkish Online Journal of Distance Education, 26(3), 128-143. <https://doi.org/10.17718/tojde.1530814>

Juárez, R., Hernández-Fernández, A., Camargo, C. B., & Molero, D. (2026). Nested learning in higher education: Integrating generative AI, neuroimaging, and multimodal deep learning for a sustainable and innovative ecosystem. Sustainability, 18(2), Article 656. <https://doi.org/10.3390/su18020656>

Khalifa, G. S. A., Elshaer, A. M., El-Aidie, S. A. M., Yee, A. S. V., Farhan, A., & Gherbi, E. A. H. (2024). ChatGPT's challenges and opportunities in higher education: A technology-user(s)-environment lens. International Journal of Management in Education, Article 140898. <https://doi.org/10.1504/IJMIE.2024.140898>

Klimova, B., & de Campos, V. P. L. (2024). University undergraduates' perceptions on the use of ChatGPT for academic purposes: Evidence from a university in Czech Republic. Cogent Education, 11(1), 2373512. <https://doi.org/10.1080/2331186X.2024.2373512>

Klimova, B., Bachmann, P., & Frutos-Bencze, D. (2025). The use of ChatGPT in academia: Perspectives of higher education students. Cogent Education, 12(1), 2508216. <https://doi.org/10.1080/2331186X.2025.2508216>

Kohnen, A. M., Newell, B., Boada, D., Breil, B. T., Fabulich, L., Miller, M., Montgomery, S. E., Mundorf, J., Poole, M., & Wusylko, C. (2025). From university to classroom: A collaborative framework for generative AI in middle school education. Middle School Journal, 56(5), 11-21. <https://doi.org/10.1080/00940771.2025.2557791>

Lee, S. C., Baby, T., Vongvit, R., Lee, J., Kim, Y. W., Cha, M. C., & Yoon, S. H. (2026). Development and validation of Generative AI Competence Scale (GenAIComp) among university students. Technology in Society, Article 103059. <https://doi.org/10.1016/j.techsoc.2025.103059>

Lee, H. Y., Chen, P. H., Lin, C. J., Huang, Y. M., & Wu, T. T. (2025). Leveraging ChatGPT for personalized reflective learning in programming education: Effects on self-efficacy, higher-order thinking, and project implementation skills. Education and Information Technologies, 30(17), 24815-24854. <https://doi.org/10.1007/s10639-025-13733-z>

Lee, S. K., Ryu, J., Jie, Y., & Ma, D. H. (2025). Motivations and affordances of ChatGPT usage for college students' learning. Media and Communication, 13. <https://doi.org/10.17645/mac.9508>

Lei, Y., Liu, J. F., Fu, X., Zhao, J. J., & Yi, B. L. (2025). The effects of a generative AI-enabled CDIO teaching model on undergraduates' computational thinking and individual psychological constructs. Computer Applications in Engineering Education, 33(5), e70075. <https://doi.org/10.1002/cae.70075>

Malik, A., Khan, M. L., Hussain, K., Qadir, J., & Tarhini, A. (2025). AI in higher education: Unveiling academicians' perspectives on teaching, research, and ethics in the age of ChatGPT. Interactive Learning Environments. <https://doi.org/10.1080/10494820.2024.2409407>

Mani, G. S., Sharma, N., Jain, R., & Pohekar, S. D. (2025). Impact of generative artificial intelligence systems on higher education in India: A Delphi study. SN Computer Science, 6(8), 910. <https://doi.org/10.1007/s42979-025-04449-5>

Martínez, C. M., Roger-Monzo, V., & Castelló-Sirvent, F. (2025). Generative AI and critical thinking in online higher education: Challenges and opportunities. RIED-Revista Iberoamericana de Educación a Distancia, 28(2), 233-273. <https://doi.org/10.5944/ried.28.2.43556>

Meng, X. P., Guo, X. G., Fang, J. W., Chen, J., & Huang, L. F. (2025). Fostering pre-service teachers' generative AI literacy and critical thinking: An RSCQA approach. Educational Technology & Society, 28(3), 202-225. <https://doi.org/10.30191/ETS.202507_28(3).TP01>

Monib, W. K., Qazi, A., & Mahmud, M. M. (2025). Exploring learners' experiences and perceptions of ChatGPT as a learning tool in higher education. Education and information technologies, 30(1), 917-939. <https://doi.org/10.1007/s10639-024-13065-4>

Namatovu, A., & Kyambade, M. (2025). Leveraging AI in academia: University students' adoption of ChatGPT for writing coursework (take home) assignments through the lens of UTAUT2. Cogent Education, 12(1), 2485522. <https://doi.org/10.1080/2331186X.2025.2485522>

Neshkovska, S., Ivanovska, L., Trajkovska, V., & Kasaposka-Chadlovska, M. (2025). ChatGPT in foreign language acquisition: A close ally or a distant concept for Macedonian university students?. Respectus Philologicus, 47(52), 50-65. <https://doi.org/10.15388/RESPECTUS.2025.47.4>

Nguyen, K. V. (2025). The use of generative AI tools in higher education: Ethical and pedagogical principles. Journal of Academic Ethics, 23(3), 1435-1455. <https://doi.org/10.1007/s10805-025-09607-1>

Nowacki, L., & Wrochna, A. E. (2025). ChatGPT theses: Identifying distinctive markers in AI-generated versus human-created texts — A multimodal analysis in university education. E-Learning and Digital Media, 20427530251331083 <https://doi.org/10.1177/20427530251331083>

O'Neill, S., Mulgrew, D., & Bagdasar, O. (2025). On the use of large language models for improving student and staff experience in higher education. Open Education Studies, 7(1), 20250086. <https://doi.org/10.1515/edu-2025-0086>

Oh, S. (2025). Evaluating mathematical problem-solving abilities of generative AI models: Performance analysis of o1-preview and GPT-4o using the Korean College Scholastic Ability Test. IEEE Access, 13, 1227-1235. <https://doi.org/10.1109/ACCESS.2024.3523703>

Oliva-Córdova, L. M., Alvarez-Icaza, I., & George-Reyes, C. E. (2025). Evaluation of generative AI use to foster critical thinking in higher education. IEEE Revista Iberoamericana de Tecnologías del Aprendizaje, 20, 237-243 <https://doi.org/10.1109/RITA.2025.3597848>

Patel, N. S., & Lim, J. T.-H. (2025). Critical design futures thinking and generative AI: A Foresight 3.0 approach in higher education to design preferred futures for the industry. Foresight, 27(2), 380-402. <https://doi.org/10.1108/FS-11-2023-0228>

Pattnaik, P. N., Pandey, S. C., & Patnaik, B. (2024). Legal education in the age of AI: Navigating the ChatGPT conundrum at Indian Law University. Emerald Emerging Markets Case Studies, 15(3), 1-13. <https://doi.org/10.1108/EEMCS-09-2024-0361>

Peláez, C. A., Munar, L. S., Galíndez, J. A. O., & Solano, A. (2026). Do generative AI assistants enhance or hinder critical thinking skills in university students?. IEEE Revista Iberoamericana de Tecnologías del Aprendizaje,  21, 19-26 <https://doi.org/10.1109/RITA.2025.3643650>

Rahiem, M. D. H. (2026). Generative AI in higher education in Indonesia: Patterns of use and learning impact. Social Sciences and Humanities Open, Article 102672. <https://doi.org/10.1016/j.ssaho.2026.102672>

Ren, J. H., Shahzad, M. F., Abbas, J., Al-Sulaiti, K. I., & Pilar, L. (2026). Factors influencing generative artificial intelligence such as ChatGPT adoption and actual use in higher education: An extended UTAUT2 perspective. Journal of Further and Higher Education, 1-23. <https://doi.org/10.1080/0309877X.2025.2573026>

Rivadeneira, L., de Luna, D. B., & Fernandez, C. (2025). Exploring the role of ChatGPT in higher education institutions: Where does Latin America stand?. Digital Government: Research and Practice, Article 3689370. <https://doi.org/10.1145/3689370>

Royer, C. (2024). Outsourcing humanity? ChatGPT, critical thinking, and the crisis in higher education. Studies in Philosophy and Education, 43(5), 479-497. <https://doi.org/10.1007/s11217-024-09946-3>

Santiago, C. M. (2025). Generative AI made me do this': Exploring the potential of ChatGPT-assisted collaborative action research in science higher education — A case in the Philippines. Educational Action Research,  1-18. <https://doi.org/10.1080/09650792.2025.2568018>

Shang, J. J., Huang, Y. H., Xu, M. X., Huang, Y., Shen, X. T., Wang, G. X., Wang, Y. T., & Zhang, L. (2025). Competition between human learners and ChatGPT: Enhancing university EFL students' reading comprehension and critical thinking through competitive questioning. Computer Assisted Language Learning, 1-35. <https://doi.org/10.1080/09588221.2025.2577356>

Sivapragasam, C., Vasudevan, M., Natarajan, N., & Saravanan, P. (2026). Exploring the challenges of LLMs in higher education: Is ChatGPT a boon or bane for the students?. Journal of Engineering Education Transformation, 39(Special Issue 2), 574–583. <https://doi.org/10.16920/jeet/2026/v39is2/26068>

Svejdarová, E. (2026). Dialogical ecopedagogy with generative AI: A postdigital framework for critical thinking and planetary justice in higher education. Cogent Education, 13(1), 2641850. <https://doi.org/10.1080/2331186X.2026.2641850>

Tai, H. Y., Lin, M. F., & Chen, Y. S. (2025). Incorporating ChatGPT into genre-based instruction for argumentative writing among EFL college students. International Journal of Applied Linguistics, 36(1), 767-781. <https://doi.org/10.1111/ijal.12777>

Tekir, S. (2026). Generative AI use in EFL writing: Associations with originality, critical reasoning, and metacognitive engagement in a Turkish higher education context. Computer Assisted Language Learning, 1-22. <https://doi.org/10.1080/09588221.2026.2617399>

Tu, Y. F., Liu, G. P., Hwang, G. J., Chen, X. W., & Guo, X. G. (2026). AI self-efficacy and knowledge graph-integrated generative AI feedback in higher education. Internet and Higher Education, Article 101079. <https://doi.org/10.1016/j.iheduc.2026.101079>

Udias, A., Alonso-Ayuso, A., Alfaro, C., Algar, M. J., Cuesta, M., Fernández-Isabel, A., Gómez, J., Lancho, C., Cano, E. L., de Diego, I. M., & Ortega, F. (2024). ChatGPT's performance in university admissions tests in mathematics. International Electronic Journal of Mathematics Education, 19(4), em0795. <https://doi.org/10.29333/iejme/15517>

Urban, M., Dechterenko, F., Lukavsky, J., Hrabalová, V., Svacha, F., Brom, C., & Urban, K. (2024). ChatGPT improves creative problem-solving performance in university students: An experimental study. Computers & Education, 215, Article 105031. <https://doi.org/10.1016/j.compedu.2024.105031>

Valdivieso, T., & González, O. (2025). Generative AI tools in Salvadoran higher education: Balancing equity, ethics, and knowledge management in the Global South. Education Sciences, 15(2), Article 214. <https://doi.org/10.3390/educsci15020214>

Vendrell, M., & Johnston, S. K. (2026). Scaffolding critical thinking with generative AI: Design principles for integrating large language models in higher education. Computers and Education: Artificial Intelligence, Article 100572. <https://doi.org/10.1016/j.caeai.2026.100572>

Waluyo, B., & Kusumastuti, S. (2024). Generative AI in student English learning in Thai higher education: More engagement, better outcomes?. Social Sciences and Humanities Open, Article 101146. <https://doi.org/10.1016/j.ssaho.2024.101146>

Wan, P., & Gu, X. Q. (2025). Designing an integrated concept mapping and generative AI approach to develop pre-service teachers' digital storytelling project outcomes and critical thinking. British Educational Research Journal, 52, 1313–1338 <https://doi.org/10.1002/berj.70060>

Wang, H. H., Da, T., Wang, Y. R., Luo, F. H., Zhang, Y. F., Tlili, A., Yang, D., Wang, Y. R., Xu, J. X., Zhu, X. X., Wan, M., & Huang, R. H. (2026). Augmentation or limitation? Generative AI's influence on college students' creative problem-solving. Thinking Skills and Creativity, 60, Article 102116. <https://doi.org/10.1016/j.tsc.2025.102116>

Xiao, S. J., & Hu, B. (2026). Exploring the relationship between ChatGPT use and students' higher-order thinking skills in tertiary education: A case study,  1-19. Education and Information Technologies. <https://doi.org/10.1007/s10639-026-13909-1>

Yigit, S., Berse, S., Dirgar, E., & Güner, S. G. (2024). Views of health sciences undergraduates on ChatGPT, an artificial intelligence-powered language model: A qualitative study. Innovations in Education and Teaching International, 62(4), 1258-1272. <https://doi.org/10.1080/14703297.2024.2391044>

Zaim, M., Arsyad, S., Waluyo, B., Ardi, H., Al Hafizh, M., Zakiyah, M., Syafitri, W., Nusi, A., & Hardiah, M. (2025). Generative AI as a cognitive co-pilot in English language learning in higher education. Education Sciences, 15(6), Article 686. <https://doi.org/10.3390/educsci15060686>

Zhan, S., & Wang, L. (2024). Integrating generative AI in higher education: Challenges, opportunities, and innovations in assessment practices within electrical engineering. Education Research and Perspectives, 51, 55-81 <https://doi.org/10.70953/ERPv51.2412003>

Zhang, M. M., & Yang, X. T. (2025). Google or ChatGPT: Who is the better helper for university students?. Education and Information Technologies, 30(4), 5177-5198. <https://doi.org/10.1007/s10639-024-13002-5>

Zhang, X., Li, D., Wang, C., Jiang, Z., Ngao, A. I., Liu, D., Peters, M. A., & Tian, H. (2023). From ChatGPT to China's Sci-Tech: Implications for Chinese higher education. Beijing International Review of Education, 5(3), 296-314. <https://doi.org/10.1163/25902539-05030007>

Zhang, Y. Y., Lai, X. M., Yi, S. P., & Lu, Y. F. (2025). Does ChatGPT-based reading platform impact foreign language paper reading? Evidence from a quasi-experimental study on Chinese undergraduate students. Education and Information Technologies, 30(7), 9737-9754. <https://doi.org/10.1007/s10639-024-13190-0>

Zhao, Y., Xu, Y., & Long, T. T. (2026). Understanding pre-service science teachers' collaborative discourse patterns in a GenAI integrated collective reflection: A network analytic approach. Journal of Science Education and Technology, 35, 141–159 <https://doi.org/10.1007/s10956-025-10241-0>

Zhou, H. X., Chen, Y. Q., Liu, Y. M., Jiang, R. J., Wang, J. Y., & Sun, M. P. (2025). Harnessing generative AI and argumentation-driven learning for entrepreneurial competence development: Evidence from university-based studies. Interactive Learning Environments, 1–18. <https://doi.org/10.1080/10494820.2025.2519122>

Zhou, R. J., He, X. L., Fan, Q., Li, Y. Y., Li, Y., Xiao, X., & Fang, J. (2025). Exploring ChatGPT-facilitated scaffolding in undergraduates' mathematical problem solving. Journal of Computer Assisted Learning, 41(4), e70077. <https://doi.org/10.1111/jcal.70077>

Total included studies: 89
